# “Bridging, brokering, and buffering”: a theoretical exploration of school leaders’ engagement with local school wellness policy implementation

**DOI:** 10.1186/s43058-020-00029-1

**Published:** 2020-05-04

**Authors:** Y. Asada, L. Turner, M. Schwartz, J. F. Chriqui

**Affiliations:** 1 Institute for Health Research and Policy, University of Illinois at Chicago, 1747 W Roosevelt Rd, M/C 275, Chicago, IL 60608 USA; 2 College of Education, Boise State University, 1910 University Drive, Boise, Idaho 83725 USA; 3 UConn Rudd Center for Food Policy and Obesity University of Connecticut, One Constitution Plaza, Suite 600, Hartford, CT 06103 USA; 4 Institute for Health Research and Policy, School of Public Health, University of Illinois at Chicago, 1747 W Roosevelt Rd, M/C 275, Chicago, IL 60608 USA

**Keywords:** School health, Implementation, Leadership theory, Focus groups

## Abstract

**Background:**

The Healthy, Hunger-Free Kids Act of 2010 (P.L. 111-296) prompted the expansion of federal requirements for local school wellness policies, which aim to improve health promoting practices across school districts in the USA. This qualitative study examined how school district superintendents—as key school leaders who are often listed as the district accountability figure for wellness policies applicable to kindergarten through 12th grade—engaged with wellness policy implementation. The inquiry was guided by evidence-informed implementation and leadership frameworks, including the Consolidated Framework for Implementation Research (CFIR) and “bridging, buffering, and brokering” strategies from education leadership theory.

**Methods:**

We conducted focus groups and interviews with superintendents (*n* = 39) from 23 states. Interviews were recorded and professionally transcribed; transcripts were team-coded in Atlas.ti v8 using an iteratively revised coding guide that was informed by CFIR, pilot testing, and during weekly analyst meetings. Principles of constant comparative analysis were employed to develop themes.

**Results:**

Most superintendents’ reported positive perspectives and personal motivations to engage with wellness policy implementation. Within the CFIR process domain, superintendents demonstrated adaptive leadership traits and employed a combination of “bridging, buffering, and brokering” strategies to lead implementation activities. Rather than focus on personal traits, an emphasis on specific strategies highlights actions that may be applied.

**Conclusions:**

The findings offer practical strategies to support superintendents with implementation, as well as a formative contribution to the dearth of theoretical frameworks in school wellness literature, particularly by advancing the specific understanding of leadership roles within a broader implementation framework. The application of education theory allowed for a deeper inquiry into the potential ways that leaders’ strategies and engagement influences implementation more broadly.

Contributions to the literature

- Research has supported the importance of leadership in advancing policy implementation but there is limited theoretical guidance in public health literature on how leaders play a role in community and school-based settings to direct or facilitate such implementation processes.
- We applied leadership-specific theory to highlight how leaders’ specific roles and strategies can advance implementation and provide insight toward a broader implementation framework (CFIR). The intentional focus on strategies offers pragmatic opportunities and removes the need for personal motivations to engage with implementation.
- These findings contribute to gaps in the theoretical development and examination of the role of leaders and strategies in the context of school wellness policy implementation. The application of a unique theory to complement CFIR provides new insights into this broader framework.

## Background

The Healthy, Hunger-Free Kids Act of 2010 (P.L. 111-296) prompted the expansion of federal requirements in the USA for local school wellness policies (hereafter, wellness policies), which are required to include provisions for physical activity, nutrition promotion and education, nutrition guidelines for all foods and beverages in schools, and other wellness-promoting activities [1]. More recently, the United States Department of Agriculture (USDA) required that—effective in the 2017–2018 school year—school districts revise their wellness policies to include additional provisions related to food and beverage marketing in schools, expanding stakeholder involvement, and updating and reporting on wellness policy assessment, amongst other requirements [2]. School districts are defined as independent special-purpose governments under local school boards and state governments. This continued U.S. federal effort to strengthen wellness policies and increase transparency about implementation highlights the ongoing need to support school districts nationwide as they fully implement and evaluate their wellness policies.

School district superintendents (hereafter, superintendents)–—as local education leaders in the U.S.—wield unique power in school districts and many school district stakeholders list them as the key accountability figure in the evaluation and reporting of wellness policies. The wellness policy final rule indicates that it is a critical time to understand how superintendents are leading ongoing wellness policies implementation and sustainability efforts; however, few studies have pursued this inquiry. Studies examining educational leaders’ perceptions of wellness policies have reported discrepancies between school board members’ confidence in school districts’ capacity to implement (46% reported being very confident), compared to state public health nutrition directors (5% reported being very confident) and wellness advocates (12% reported being very confident) [3]. Another study examined school board members’ perceptions about improving school food environments through nutrition policy [4]. Neither study was specific to superintendents, nor to implementation experiences since the most recent federal final rule. To our knowledge, no studies have focused specifically on the unique characteristics or activities of superintendents with respect to wellness policy implementation, and importantly, no studies have applied evidence-informed frameworks to guide this inquiry.

The current work seeks to fill this gap by explicitly examining the role of district leadership—at the level of the superintendency—in leading wellness-related changes. While building-level leadership is also crucial, there is reason to believe that the vision of a superintendent can impact an entire district, and the current work sought to also examine the specific strategies that successful superintendents use. The objective of this study was to apply a theory-driven framework to understand the characteristics of and strategies utilized by superintendents for wellness policy implementation.

### Complementing frameworks: implementation science and educational leadership

This study of health policy intervention in education agencies was grounded firmly within the scholarship in the disciplinary areas of implementation science and education leadership. The former—Consolidated Framework for Implementation (hereafter: “CFIR”) within implementation science—allowed for an overarching framework in which to understand the broader context of wellness implementation. The latter—educational leadership theory—provided a micro-framework embedded within CFIR to narrow in on the specific strategies applied by leaders during implementation phases. Our intention was to apply the leadership framework to inform the detailed processes and strategies employed by leaders that the overarching implementation (CFIR) framework did not address specifically. Each framework is described next.

### Implementation science

CFIR is one of many frameworks in the growing field of implementation science that offers evidence-informed guidance for understanding the processes yielded by the implementation of programs and policies in complex, dynamic, and hierarchical settings [5]. As indicated by its name, the CFIR is a theoretical framework designed to synthesize existing models, theories, and frameworks in existence prior to its publication in 2009. Notably, the majority of research using the CFIR has been conducted in the field of health services; however, its development process also included seminal work from implementation scholars in the education domain such as Fixsen and colleagues [6]. Although focused more on health interventions, the comprehensive nature of the CFIR offers an approach for conceptualizing key domains that are relevant to school wellness. The CFIR identifies 5 core domains: outer setting; inner setting; process; individuals involved; and intervention. The constructs of “leaders and leadership” are mentioned across domains in several interrelated constructs [5]. For example, leadership constructs are included in the inner setting in “learning climate,” “readiness for implementation,” and “individual identification with organization” (implementation leaders). In addition, leadership is prominent in the “process” domain, due to the demonstrated importance of leadership in creating systems change [5]. Table 1 lists the four types of implementation leaders described within the process domain. The widespread inclusion of these constructs throughout the CFIR domains highlight the critical importance of leadership in advancing (policy) implementation. However, while the types of leaders suggests who the leaders are and some examples of their strategies, the CFIR itself does not identify specific leadership strategies or relationships to other constructs, resulting in difficulty applying the framework to phenomenon such as implementation of wellness initiatives. Thus, using CFIR as an overarching framework, we incorporated a more specific set of theories—described next—to narrow in on such an inquiry and to address the aforementioned limitations with using the CFIR alone to study leadership and its relationship to implementation.

**Table 1:** CFIR process domain: implementation leader typologies [5]

Leader	Definition
Opinion leader	Leaders with “formal or informal roles and influence on attitudes and beliefs of their colleagues with respect to implementation”
Formally appointed internal implementation leaders	Leaders “formally appointed with responsibility for implementing an intervention as a coordinator, project manager, team leader, or similar role”
Champions	Leaders “who dedicate themselves to supporting, marketing, and driving through” an implementation; “overcoming indifference or resistance that the intervention may provoke”; differentiated from Opinion Leaders as Champions are more actively associated with supporting the intervention
External change agents	Leaders from an outside entity “who formally influence or facilitate intervention decisions in a desirable direction.”

### Education leadership theory

**Fig. 1:**
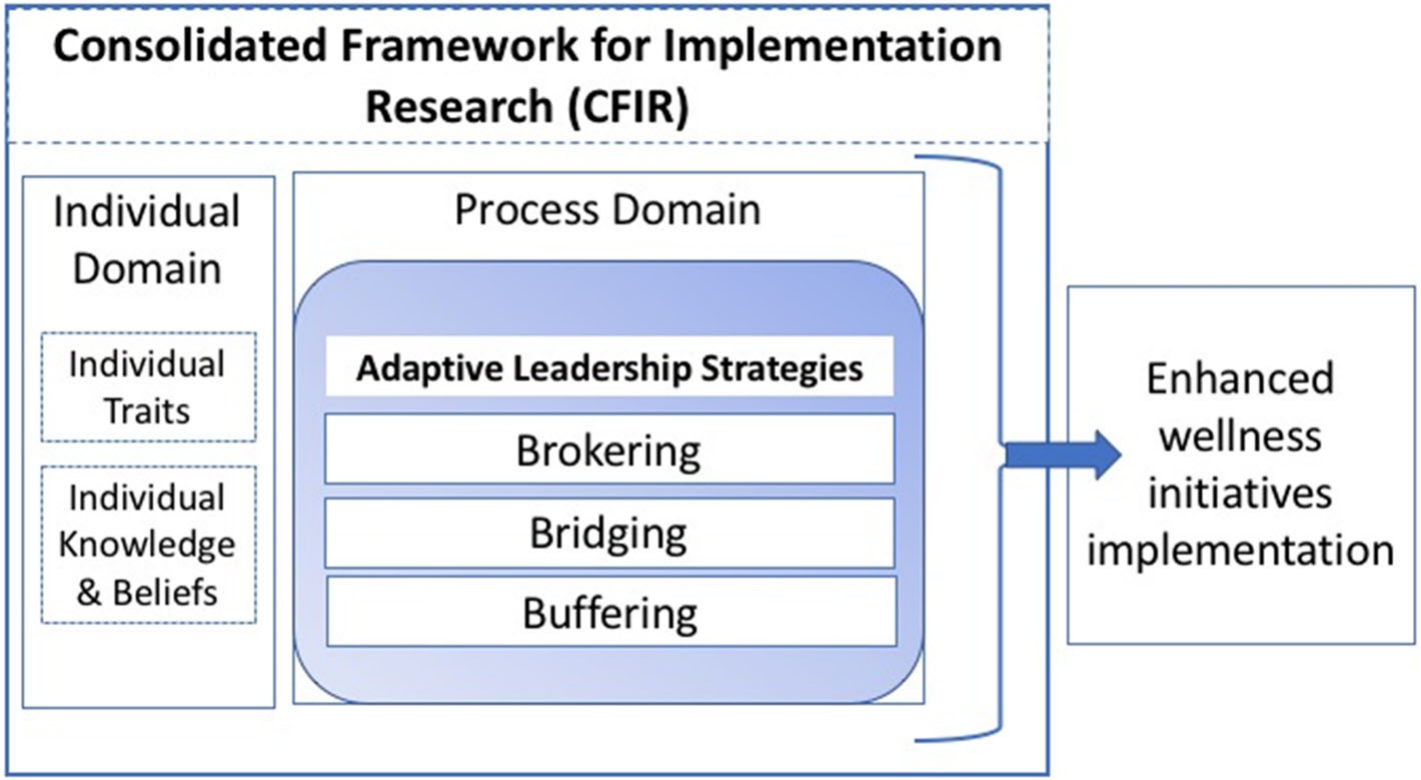
Educational leadership theory embedded within the Consolidated Framework for Implementation Research (CFIR) framework. The framework highlights CFIR domains—individual and process domains—as an overarching framework; the adaptive leadership strategies are embedded within the process domain.

Education has long recognized the importance of leadership in promoting system-wide change that translates into improvements in school-level instructional practices [7–10] and student outcomes [11, 12]. In terms of examining how implementation happens—that is, the process of change—we found the framework of adaptive and proactive leadership, as well as their affiliated strategies of “bridging, brokering, and buffering,” identified by education scholars helpful in characterizing the different types of strategies used by district leaders [13, 14]. Other work has extensively studied these strategies and found them crucial among school district leaders who “craft coherence” towards effective top-down and bottom-up mechanisms to improve implementation. *Adaptive leaders* “recognize that one or two persons located at the top of the organizational hierarchy are unlikely to know all that they need to know and do all that is required to address complex, novel, and uncertain problems….and they distribute it to others situated lower on the organizational hierarchy,” while *proactive leaders* anticipate change and foster organizational capacity and readiness [13]. It is understood that effective leaders may utilize both styles, along with three broad strategies: “bridging, brokering, and buffering.” *Bridging* strategies refer to leaders’ ability to create networks, establish “boundary-crossing” activities, and facilitate communications, all with the goal of advancing organizational goals [13]. As school district leaders, superintendents have unique authority to represent the district as they reach across “boundaries” to make such connections. *Brokering* strategies involve leaders’ ability to adapt the policy to the school district, by translating policy language into shared practices and vocabularies. A key activity is creating common purposes and ensuring that stakeholders are buying in to implementation activities [13]. Lastly, *buffering* strategies work to minimize or prevent conflict, thereby facilitating implementation activities; this may include strategically allocating or removing time and resources from activities that do not directly meet school goals towards implementation [13]. Within the CFIR process domain, we applied the conceptualization of “bridging, brokering, and buffering” strategies to describe the process of *how* these superintendents implemented wellness policies. Figure 1 illustrates the combination of frameworks.

As noted, the goal of this project was to examine the role of superintendents in promoting the implementation of wellness policies. We framed the study within the overarching framework of the CFIR as well as a micro-framework of leadership traits and the strategies of “bridging, brokering, and buffering”. Building upon these theories, the objective of this study was to understand how superintendents, as key school district leaders, influenced wellness policy implementation processes through their individual traits and applied strategies.

## Methods

### National Wellness Policy Study design and methods

The National Wellness Policy Study is a mixed methods study that examines the implementation of Healthy, Hunger-Free Kids Act of 2010 and its related policies [15]. The qualitative component included a series of stakeholder focus groups and interviews with food service directors, high school students, superintendents, and parents of middle-school students. Broadly, stakeholder interviews focused on experiences and perspectives related to implementation of wellness policies and nutrition standards, with the overarching aim of providing policy- and practice-relevant findings for school stakeholders, local, state and federal government agencies, and school and health advocates. Additional findings from the superintendent and other stakeholder studies are described elsewhere [16–18]. The study was approved by the University of Illinois at Chicago Institutional Review Board (#2015-0720) and University of Connecticut Institutional Review Board (#H15-165).

### Participants

The current study focuses on one component of the superintendent study mentioned above, which included focus groups and key informant interviews with superintendents and assistant superintendents (hereafter referred to together as superintendents) who attended The School Superintendents’ Association (AASA) annual meeting during March 2017 in New Orleans, LA. AASA is a professional organization that includes over 13,000 superintendents, chief executive officers, and senior school administrators [19]. Eligible participants were superintendents registered for AASA’s annual meeting, currently employed at any level of public K-12 school district, and English speaking. We sent email invitations to participants who had registered for the AASA meeting to attend one of six focus group sessions. Participants who responded were sent a consent form with further information about the study to review before the focus groups. We assigned participants to a focus group based on their school district characteristics, to attempt to create “homogenous” groups in order to facilitate discussions [20]. Since participants were traveling from many states across the USA, it was not possible to establish relationships prior to the focus groups.

### Instruments and data collection

We developed a focus group guide based on the research questions and revised after pilot testing with two superintendents to refine the flow and appropriate terminology. The guide asked questions broadly about superintendents’ awareness of wellness policies, oversight and evaluation, technical assistance and resources, perceived benefits and barriers, and food and beverage marketing policies. We developed a follow-up interview guide after focus group analysis was underway to reflect additional topics that emerged, as well as to ask more in-depth experiences with implementation. The follow-up guide was not pilot tested due to time constraints. Both guides are available upon request to the corresponding author.

Focus groups were conducted in a meeting room during the AASA meeting and lasted approximately 60 min. Each focus group had between 5 and 7 participants, for a total of *n* = 39 participants. Focus group participants were asked to participate in follow-up interviews after the meeting. Those who agreed were contacted for interviews (*n* = 14) over the telephone; interviews lasted 40–60 min. Both were conducted by trained qualitative researchers (JC, YA); room assistants took notes and oversaw administrative tasks. After each focus group and after telephone interviews, moderators and assistants debriefed initial insights and discussed revisions to the instruments. Both researchers have extensive experience leading qualitative research projects and conducting qualitative focus groups and interviews. Participants completed a brief survey that included questions about their demographics, awareness, and engagement with their school district’s wellness policy activities such as implementation and reporting. Superintendents were sent a $50 Amazon gift card following the focus groups.

### Data coding and analysis

Focus groups and interviews were audio recorded, transcribed, and uploaded into Atlas.ti Qualitative Data Analysis Software v8 for team coding. Transcripts were not returned to participants prior to data coding and analysis. An a priori coding guide was developed, based on study questions, and iteratively revised throughout weekly team coding meetings. Three analysts met to discuss discrepancies to coding, revisions to code meanings, and emergent themes. Memos were used to document progress, study decisions, and themes [21, 22]. Matrices of themes for the focus groups and follow-up groups were compared to document theme trends, as well as new themes from the follow ups. Analysts paid attention to focus group discussions that suggested consensus or overall agreement and compared and contrasted these themes to individual interview data. When outliers or uncommon perspectives were observed, these were documented in memos. Individual interviews also contained additional themes that built upon and provided further details to the focus group topics. Analysts discussed thematic saturation as analysis and writing progressed [23]. Atlas.ti v8 exploratory functions were used throughout analysis to confirm/disconfirm trends and further deepen the analysis. Due to time constraints, participants did not provide feedback on emergent themes prior to “finalization” of findings [24].

## Results

Superintendents from all four Census regions attended the focus groups, with a majority employed in suburban school districts (54%); in small school districts (72%); and in school districts with a majority of White students (64%). Table 2 lists the characteristics of the school districts where the superintendents worked.

**Table 2:** Characteristics of superintendents’ school districts

Characteristic	*N* (%)
Census region	
West	6 (15%)
Northeast	15 (38%)
South	7 (18%)
Midwest	11 (28%)
Locale	
Rural	9 (23%)
Suburb	21 (54%)
Township	6 (15%)
Urban (large- to mid-size city)	3 (8%)
Socioeconomic status (tertiles)	
Low (0–33%)	19 (49%)
Medium (34–66%)	12 (31%)
High (67–100%)	8 (20%)
District size (tertiles)	
Small (≤ 5312)	28 (72%)
Middle (5313–10,624)	9 (23%)
High (≥ 10,625)	2 (5%)
Race/ethnicity	
Majority White	25 (64%)
Majority Hispanic	3 (8%)
Majority Black	4 (10%)
Other	7 (18%)

Broadly, superintendents expressed positive perceptions about expanding wellness efforts in their school districts. Our findings reflect individual characteristics, leadership traits, and strategies applied by superintendents from their first-hand accounts of wellness policy implementation. Participants employed adaptive leadership traits and applied combinations of "buffering, brokering, and bridging" strategies, indicating there was not a *one size fits all* approach to their involvement. Notably, leaders in larger school districts employed different leadership traits compared to those in smaller school districts. Table 3 lists additional illustrative quotes that support the study themes.

**Table 3.** Additional themes and illustrative quotes

Theme	Definition	Illustrative quotes
Change agents/implementers		If we emphasize working with the whole child, take a holistic educational approach that we’re in it for the kids. As long as we remember that, we’re mindful of how important, how profoundly important, student health is to outcomes but also how central it is to our mission. All of our districts have mission statements that talk about lifelong learning and productive members of society. If people are morbidly obese, or they can solve a complex mathematical equation but they can’t take care of themselves because we didn’t instill those habits, then we’ve fallen terribly short of what we need to do as educators. I think the more data you can provide as evidence…but to be perfectly honest, who doesn’t already know that? I mean, sorry, but are we really doubting that healthier kids do better [academically]?….
Adaptive leaders	“Recognize that one or two persons located at the top of the organizational hierarchy are unlikely to know all that they need to know and do all that is required to address complex, novel, and uncertain problems….and they distribute it to others situated lower on the organizational hierarchy”	I think it’s more of a team approach. The district nurse has her part, business manager has another part and the teachers have another part because they’re implementing the standards within the classroom setting. So there’s not like a person doing all of it. It’s a very differentiated approach. It’s always the superintendent but we have people…there’s certainly layers or levels that help us out. I would say cabinet member officials as well as assistant superintendent that really do the groundwork of everything. Yes, if it’s not implemented correctly, it falls on my shoulders.
Bridging strategies	Leaders’ ability to create networks, establish ‘boundary-crossing’ activities, and facilitate communications, all with the goal of advancing organizational goals	We’ve partnered with a Let’s Move kind of organization. We have a local organization in our city, so the elementary district has partnered with that group to try to increase physical activity for the community, but focusing on starting in schools.
Brokering strategies	Leaders’ ability to adapt the policy to the school district, by translating policy language into shared practices and vocabularies	…All strata of personnel are aware of the policy…if they don’t know it, they can’t implement it, so I take responsibility.’
Buffering\ strategies	Leaders’ ability to minimize or prevent conflict, thereby facilitating implementation activities; this may include strategically allocating or removing time and resources from activities that do not directly meet school goals towards implementation.	I say there are three things I’m focusing on and that’s it. One of them will always be the wellness piece. That message is reiterated over and over and over again [in reference to bully-pulpit].

### Individual characteristics: “Doing what’s right for the children”

A key CFIR domain for understanding implementation pertains to examination of the characteristics of the individuals involved, with a focus on the dynamic interplay between the individual and organization [5]. The importance of this relationship in school districts cannot be understated, given the power held by superintendents. Within this domain, a number of personal attributes and traits are identified as being crucial for implementation success, including: “tolerance for ambiguity, intellectual ability, motivations, values, competence, capacity, innovativeness, tenure, and learning style” [5]. Several superintendents in this study demonstrated a high degree of personal interest in wellness; for example, as former physical education teachers or coaches, or expressed strong motivations—centered around “doing what is right for the children*”*—towards improving wellness environments.

*Personally, as a superintendent, I love economics and I know it’s all about money, the bottom line. However, philosophically, I personally have to do what’s right for children. To turn it from red to black because we’re losing money, I feel a personal obligation to spin it and say to the parents ‘we’ve got to take care of your kids holistically, so they can think and become responsible citizens and have a responsible life.*

While superintendents’ role requires an attention to the *economics* of running school districts, participants’ personal values of attending to the holistic needs of students, including their health needs, bolstered their support and engagement with implementation activities. The superintendents in this study demonstrated such strong assertions of personal values and motivations to serve the “whole” student in this way.

### Individual characteristics: knowledge and beliefs

Also, crucial in the individual domain in CFIR are the knowledge and beliefs of implementers with respect to the policy or intervention. Whether based on “objective” knowledge of the intentions of the policy, or “subjective” opinions based on personal or peer feedback, this type of content knowledge—and beliefs about it—can directly influence an individuals’ involvement with implementation. Almost all superintendents reported perceptions that wellness policy implementation had the potential to improve academic performance, which is a primary goal amongst school leadership [25]. Notably, these two traits within individual characteristics may be expected from a “champion” leader type (Table 1). This common perception influenced superintendents’ engagement with implementation:

*We have tried to message, repeatedly and routinely over the years around student health, that there are logical positive correlations between proactive health behaviors and student achievement*, *and that cognitive performance is often a reflection of student health. And, therefore, it is the business of schools to be involved in physical activity, nutrition literacy and other health supports for our students to ensure that they are successful, that every student thrives.*

Superintendents’ shared beliefs that attention to health positively impacts academic performance highlighted a key reason for their motivations to engage with and provide leadership around wellness initiatives. As the superintendent states above, if one believes in this relationship, then the next step is to make health supports and subjects like nutrition literacy *the business of schools.* Such a shift in perspective that includes wellness within their purview of improving academics thus influenced superintendents’ engagement with wellness implementation.

### The process domain and adaptive leadership traits: “while you need to lead it, you do not have to do it”

The CFIR process domain considers implementation to occur over a series of sub-processes and formal/informal activities that are refined and re-evaluated over the course of time; as described, the types of leaders that influence these activities are considered within the process domain (Table 1). Beyond the types of leaders, educational leadership frameworks guide an inquiry of how those leaders specifically engaged with implementation with a consideration for leadership strategies. Superintendents in this study exercised adaptive leadership, in that a *differentiated approach* or delegation of tasks was noted to be critical to their approach. Notably, study participants in larger school districts reported the ability to delegate, while those in smaller districts reported having less staff capacity and thus were involved more with implementation activities. Table 4 lists the common roles delegated for wellness policy activities for larger school districts. A key structure with which this adaptive approach was executed was the wellness committee, which allowed for superintendents to provide oversight over a centralized group of stakeholders and delegated activities.

*I think most superintendents I can think of understand the significance of wellness in their districts…One of the things I offer to my colleagues is the understanding that, while you need to lead it, you don’t have to do it…For me, that’s the most important part of our wellness policy. Who is the coordinator and that the [wellness] committee is functioning.*

**Table 4.** Common district and school positions involved in wellness policy implementation

District level	School level
• Child nutrition directors • Assistant superintendents • Director of nurses • Office of student and staff wellness • Human resource directors • Student services supervisors • Business managers • District wellness coordinators • Communications directors • Supervisors of special services	• Principal/administrator • Cafeteria managers • School nurses and psychologists • Guidance counselors • Health and physical education teachers • Other teachers

In part, the superintendent offers this reassurance for fellow district leaders who may be overwhelmed with the many competing priorities inherent in their role. This reassurance also underscores the importance of the infrastructure needed to ensure that the many implementation processes are delegated and executed in an effective manner. The wellness committee provided a natural structure for the superintendent to oversee these activities. In addition, a key role in the translation of policies to practice was noted to be school principals, who ensured consistency of messaging and implementation at the school level:

*The buck stops with the principal. We have assistant principals and principals, so building administrators work hand in hand with the staff. So it’s kind of a group effort, but the buck stops with the principal.*

While the superintendent oversees school district-wide implementation, school-level principals serve a critical role in working directly with staff and students and ensuring implementation processes are completed. Thus, the superintendent reminds us that in addition to the infrastructure of the broader district wellness committee, school principals are a key implementation figure.

### Adaptive leadership and “bridging” strategies: building external partnerships to enhance resources

Adaptive leaders may employ bridging strategies such as “boundary-crossing activities” that connect the organization to other entities to enhance implementation. Superintendents played a key role in creating or facilitating external partnerships which enhanced the organizational capacity of the school district to implement wellness policy initiatives. While some participants were directly involved with forming partnerships, others noted that they facilitated this activity by delegating to others; importantly, the superintendents’ direction was key to securing these relationships. Many participants noted that partnerships enhanced the districts’ access to both monetary and informational resources for implementation, allowing wellness initiatives to be advanced in a way that the school district alone could not execute. For example, partnerships that led to the successful attainment of state or non-profit/private grants for cafeteria equipment facilitated the provision of healthy salad bars, thus improving access to fresh fruits and vegetables. While many of the provisions can be implemented without additional costs, some initiatives—such as cafeteria or physical education equipment—may require districts to obtain additional funding. Further, informational resources were also noted to be critical; superintendents liked that partners provided evidence-informed resources that aligned with the goals of the school districts’ wellness policies.

*We’ve also worked with one of the state universities. They have an outreach program, especially for the parent education, with nutrition and physical activity.*

This strategy of enhancing resources was critical since some superintendents described challenging budgetary times. Obtaining external resources and financial support was critical to advancing implementation efforts.

### Adaptive leadership and “brokering” strategies: creating an aligned vision

Adaptive leaders may employ brokering strategies such as creating an aligned vision for the implementation changes within the organization through formal and informal influence. Superintendents directly facilitated implementation by translating the wellness policy into common messages and creating a district-wide “aligned vision” for wellness initiatives. Through various communication activities, coordinating, and framing helped to resolve myths and indicate to school stakeholders that wellness initiatives were a priority coming from district leadership. Described by one participant as "*putting feet to the policy*," these activities were key to ensuring that implementation processes went smoothly.

*I see my role as being able to show people the gap between what our policy says and our actual practice. Helping us find ways to close that. So celebrating what we are doing well but also finding the one or two priority areas we need to work on further.*

The superintendent has incredible power in the school district to identify and communicate *priority areas* that direct the activities of school stakeholders. Their attention to closing “*the gap”* between policy and practice influences the actions of those responsible for implementation tasks and moves the processes along in a smoother fashion.

### Adaptive leadership and “buffering” strategies: “taking on the naysayers”

Adaptive leaders may employ buffering strategies such as “prevent[ing] conflict and easing pressures” that may arise during implementation. Superintendents described the importance of such strategies, particularly because their public attention to and support of wellness initiatives was critical to garnering buy-in from school and community members. Their support eased any potential push-back from those less supportive of the policy (i.e., *the naysayers*). Participants described their support through maintaining the “bully pulpit” and therefore a focused attention on implementation.

*So in the background, sometimes I have to take on the naysayers to someone who feels that the wellness initiatives stifle their parenting responsibilities. That doesn’t happen very often, but it has happened so it’s worth noting that at some point, I become the defender of the policy. I try to do that in the background though.*

In some cases the role of being “protector” of the policy was one of enforcing power more directly. For example, one superintendent described the limited power of food service directors to enforce requirements, thus necessitating intervention.

*Food service directors generally don’t have the same kind of teeth in their recommendations to principals…I have the luxury in my role of saying, ‘I appreciate that you don’t like this, but this is what we’re going to do and you’re going to have to do it. And how can I help you do it or can I send the food service director over to help you understand how to do it.’*

This important strategy by the district leader highlights the hierarchical nature of school districts and explains why superintendent support for the wellness policy is commonly listed as a critical facilitating factor for implementation [16]. In this way, superintendents used both powers of “defender” or “protector” of the policy, or a more authoritative power of their administrative role to influence other stakeholders’ acceptance and buy-in for implementation changes. In either case, their ability to “buffer” any challengers played a critical role in ensuring that barriers were addressed and implementation processes progressed.

## Discussion

This study is the first—to our knowledge—that offers a theoretical examination of the role of leadership characteristics and strategies in the context of school wellness policy implementation. We understand these leadership constructs of “bridging, brokering, and buffering” as embedded within—and providing insight toward—a broader implementation framework, the CFIR. As we expected, wellness policy implementation is a dynamic process, involving many levels of influence within the CFIR, but importantly, adaptive leadership constructs provided more detailed insights into the CFIR individual characteristics domains. As noted by the CFIR authors:

People are not passive recipients of innovations. Rather….they seek innovations, experiment with them, evaluate them, find (or fail to find) meaning in them, develop feelings (positive or negative) about them, challenge them, worry about them, complain about them, “work around” them, gain experience with them, modify them to fit particular tasks, and try to improve or redesign them often through dialog with other users [5] (p 598).

The individual characteristics domain was salient in this study, with common motivations expressed as the “betterment of children’s lives” and improved academic performance through health and wellness as important facilitators. This finding regarding the moral imperative of improving children’s lives is highly consistent with seminal work in education regarding the importance of “moral purpose” as an essential characteristic of cultural change leaders [7]. As noted by Fullan (2002), “moral purpose is social responsibility to others and the environment. School leaders with moral purpose seek to make a difference in the lives of students” [7]. The current work re-iterates the central importance of that personal characteristic among district leaders who prioritize changes to improve the health of students.

While individual characteristics and motivations for wellness were strong facilitators in this study, we stress that this may be unrealistic to expect from the broader population of superintendents. Instead, a more helpful emphasis may be on the specific strategies that were employed, which highlights actions that can be learned through adaptive leadership strategies, rather than personal traits, which are less malleable. At this formative stage in our inquiry, there did not appear to be a one size fits all or optimal combination of strategies, indicating that superintendents may utilize any or all that are feasible in their respective roles. Notably, this finding provides a more nuanced understanding of leaders' roles, documenting that superintendents could both be formal “opinion leaders” as well as “champions” (Table 1) depending on the situation. These action-oriented strategies of “bridging, brokering, and buffering” provide important evidence-informed recommendations for child health and wellness advocates and government bodies providing technical assistance to school leaders as they implement the revised provisions of their wellness policies.

In addition, leaders in larger school districts emphasized the importance of demonstrating adaptive leadership and relying on experts across their districts to implement the many components of the wellness policies. Further, as demonstrated by adaptive leaders, superintendents employed a combination of “bridging, brokering, and buffering” strategies to support implementation in a range of activities. This is consistent with previous educational studies that document adaptive leaders’ use of these three overarching strategies to effectively facilitate policy implementation [13].

### Limitations

Several limitations to the study are notable. The majority of superintendents were well-engaged or motivated to engage with implementation (a handful had not engaged but were curious about wellness or were still in the early stages); participants were not meant to be representative of the larger population. While their higher level of engagement was not determined a priori as eligibility criteria, it offered “information-rich” data from those who had previous experience with implementation [21]. Future research may examine less engaged superintendents for additional insights and differing perspectives. In addition, the sample of superintendents were employed more heavily in suburban districts with few urban or rural districts; this likely influenced our findings given that school district size was noted to be an important factor in their strategies. Further, this study was a qualitative examination based on superintendent perspectives at one point in time. We did not measure any behavioral or environmental outcomes from implementation, and thus did not triangulate superintendent accounts of implementation with measured changes in school wellness environments.

### Implications

School districts nationwide continue to implement wellness policy provisions to comply with the recent final rule and updated requirements effective school year 2017–2018. From a practice perspective, these findings provide formative theory-driven strategies and “first steps” for how technical assistance providers can encourage increased wellness engagement amongst superintendents. The focus on strategies—rather than individual traits—aims to encourage a wider range of superintendents than the smaller number who may be inherently interested in or motivated by health and wellness. Advocates and technical assistance providers can encourage superintendent engagement in the following ways: (1) assist with the formation or ongoing support for wellness committees to provide an infrastructure for superintendents to oversee, rather than personally taking on all wellness initiatives; (2) educate superintendents directly on the goals of the wellness policy, including links to the potential to improve student academic performance; (3) provide resources about potential partnerships with neighboring nonprofits, universities, and other technical assistance providers.

The leadership-specific constructs presented in this study elicited several data-driven insights into the overarching CFIR framework. For example, involvement of formally appointed leaders are identified in the process domain as important to facilitate implementation (Table 1); our findings suggest this may be due to effective brokering strategies, wherein superintendents facilitate a “shared vision” for the policy through buy-in from stakeholders. In another example, the CFIR “inner setting” domain, readiness for implementation is an important construct, with leadership engagement, available resources, and access to information and knowledge as key sub-constructs. This study highlights how superintendents’ bridging strategies enhanced access to both resources and information, linking these three constructs together. Taken together, this targeted examination of education leadership theory provides further insight into how the constructs may operate within the broader CFIR framework.

This formative application of leadership theory to wellness policy implementation also offers implications for future research. Our study indicates that superintendents in small and less resourced school districts experienced barriers to delegate implementation tasks. Future research may further examine the relationship between leadership strategies, individual constructs, and school districts characteristics. For example, can leadership strategies compensate for these barriers posed by school size and resources? Or on the other hand, do characteristics like small district size facilitate the application of some strategies? In addition, our examination only begins to link leadership constructs with CFIR domains and sub-constructs; we intend for the strategies identified here to be a starting point for discussion about such relationships.

## Conclusion

This project contributes to the nascent literature on superintendent leadership traits and employed strategies in implementation of wellness policies, using a theory-driven leadership framework. While being mindful of the stated limitations, we provide a formative but focused examination of education leadership strategies as embedded within a broad CFIR framework. The specific strategies allow for considerations for technical assistance providers to be relevant for a broader superintendent audience who may not have such inherent personal interests or strong motivations to work in wellness. We intend for this formative theoretical work to be a starting point for discussion and further empirical inquiry amongst school leadership and school wellness research communities.

## Data Availability

The datasets generated and/or analyzed during the current study are not publicly available due to institutional review board regulations but selective deidentified and aggregated data may be available from the corresponding author on reasonable request.

## Abbreviations

AASA: The School Superintendents Association
CFIR: Consolidated Framework for Implementation Research
